# Quantitative Assessment of Biomechanical Deviations in Hybrid III Dummy Response Caused by Accessory Lumbar Supports

**DOI:** 10.3390/s25247647

**Published:** 2025-12-17

**Authors:** Wanda Górniak

**Affiliations:** Laboratory of Vehicle Dynamics and Safety, Department of Automotive Engineering, Mechanical Faculty, Wrocław University of Science and Technology, Na Grobli 13, 50-421 Wrocław, Poland; wanda.gorniak@pwr.edu.pl

**Keywords:** lumbar support, lumbar overlays, rear impact, sled test, Hybrid III dummy

## Abstract

Rear-end collisions remain a significant category of road accidents, despite widespread passive safety systems. Although modern seats are designed to reduce injury risk, the influence of accessory lumbar supports on passenger safety is still insufficiently investigated. This study analyzes the biomechanical response of a Hybrid III 50th percentile dummy on a vehicle seat fitted with various lumbar support types, compared to a reference configuration. Tests were conducted on a sled bench, simulating impacts of varying energy using crash pulses of 10 g, 15 g, and 20 g, for each tested lumbar support configuration in carefully controlled laboratory conditions. A key element of the procedure was analyzing changes in head and chest acceleration waveforms relative to results obtained for the reference seat. To quantitatively assess discrepancies between signals, the Root Mean Square Error (RMSE) and the CORA (CORrelation and Analysis) objective rating method were applied. These tools enabled precise separation of amplitude changes from phase shifts arising from different system dynamics. The results show that additional equipment elements modify dummy–seat interaction, with the extent of biomechanical response changes also depending on crash pulse value. This indicates that ergonomic supports are not biomechanically neutral and should be considered in comprehensive safety analyses.

## 1. Introduction

Currently, rear-end collisions are increasingly recognized as one of the most hazardous road traffic incidents. Although modern vehicles are equipped with advanced passive safety systems that are effective, particularly in frontal and side impacts, rear-end impacts remain problematic. In such situations, occupants rely heavily on the design of the seat and the head restraint. While airbags, seatbelt pretensioners, and crumple zones activate during frontal and side impact crash pulses, in rear-end collisions, a properly designed seat and a correctly adjusted head restraint constitute the “last line of defense”, limiting the risk of whiplash injuries and the resulting Whiplash Associated Disorders (WAD). Whiplash injuries are difficult to predict and may be associated with long-term clinical consequences of WAD [1,2,3]. In a rear-end collision, the motion occurs sequentially: first, torso translation occurs as the seat pan and seatback “push” the pelvis and torso forward relative to the head, which lags behind due to inertia, while seatback deflection increases [4]. This leads to the straightening of the thoracic kyphosis and compression injuries of the cervical spine; subsequently, a characteristic “S-shape” curve forms in the cervical spine, with the lower segments in flexion and the upper segments in extension, representing a particularly sensitive moment for ligaments and intervertebral joints [4,5]. This is followed by the head contacting the head restraint, which, if delayed or accompanied by excessive seatback deflection, increases the relative velocity of the head at the moment of initial support [6,7]. Contact delay is critical because the longer the head remains unsupported, the greater the relative head-to-torso motion becomes, which increases the NIC/Nkm (Neck Injury Criterion/Neck protection criterion) values; simultaneously, the risk of non-physiological rotations in the C5–C7 segments rises [6,8,9]. Finally, the extension and rebound phase occurs, where the elastic energy stored in the seatback, combined with muscle activity, may exacerbate the rebound motion and generate secondary moment peaks [4,10]. Although whiplash injury is typically associated with rear-end collisions, it has been established that this injury mechanism can also occur in frontal impacts, particularly when the occupant travels with a reclined seatback and during a low crash pulse; the greater the seatback recline, the higher the accumulation of compressive forces in the cervical spine, and the resulting mechanism may resemble that observed in rear-end collisions [11,12].

Rear-end collisions are typically analyzed under conditions of a relatively low crash pulse, based on the assumption that in such situations the speed differences between vehicles moving in the same direction are small [13,14,15,16]. In controlled experiments, the crash pulse rarely exceeds 11–12 g, which is consistent with most regulatory and research protocols. Such simulated events are intended to reflect typical road situations with minor speed discrepancies, characteristic of congested urban areas. A change in velocity (Δv)in the range of 15–20 km/h corresponds to such impacts and is often associated with whiplash injuries, even in the absence of structural damage [1]. Biomechanical studies confirm that even at relatively low Δv values, neck extension and the rebound phenomenon generate significant inertial loads in the cervical spine [10,17]. Observational data from clinical and road accident databases support these findings, indicating a high incidence of soft tissue injuries in low-speed rear-end collisions [18,19]. Newer laboratory studies emphasize the necessity of extending rear impact tests to acceleration levels exceeding 15 g to account for conditions observed in more severe collisions [10,20,21,22]. Experimental results indicate that at such crash pulse amplitudes, the probability of cervical spine overloading and early rebound increases significantly, especially when the structural stiffness of the seatback is high and the energy dissipation capacity is limited [18,23]. Kinematic observations additionally show that rebound velocities exceeding 6 m/s may occur shortly after impact, which increases loads on the upper torso and the cervical spine [24,25].

In controlled test conditions, head accelerations in rear impacts are, on average, about three times higher than in frontal collisions, and volunteers rate these events as more traumatic. Kinematic analyses reveal complex neck motions and significant differences in the time to head restraint contact; a high risk of neck injury is associated with a delayed head restraint response and excessive seatback compliance when the torso accelerates faster than the head [6,11,26,27]. Against this background, the characteristics of the seat and head restraint take on particular importance. Progress in the protection of rear seat occupants still lags behind that for front seats due to a smaller number of active elements [28]. Greater compliance of the back and pelvis supports shifts the torso center of mass rearward and increases the range of neck extension; therefore, a balanced relationship between cushioning and motion control is required [29]. An overly soft head restraint worsens indices, whereas increasing its stiffness reduces moments, shear forces, and injury metrics by approximately 15–25% [30]. An excessively compliant soft seat prolongs the time to head restraint contact and increases the amplitude of neck displacements, which argues in favor of materials with gradient stiffness or adaptive structures [31,32]. Any additional layer increasing the effective head-to-head restraint distance and reducing support hardness raises the NIC and Nkm values, while reducing the backset lowers the values of injury criteria [8,9]. From the perspective of system geometry, the target backset should be less than 50 mm at the moment of impact, accounting for the deflection of the padding and the seatback [7].

Furthermore, the system response is influenced by population characteristics and posture. Females experience higher head and neck accelerations and earlier contact with the head restraint than males, which is associated with anthropometric differences and body positioning; soft seatbacks increase the rearward displacement of the pelvis and torso, exacerbate neck flexion, and raise the NIC, while rigid seatbacks limit motion but increase peak accelerations [8]. Excessively stiff seat pans and a lack of seatback compliance raise the risk of cervical loads, whereas moderately elastic seats may limit early compression [33]. A slight increase in horizontal backset increases head displacement and rotation by 10–20%, and an increase in vertical distance raises flexion accelerations by approximately 15%; seats with controlled deformation, such as WHIPS (Volvo’s Whiplash Protection System), reduced the risk of WAD by 30–40% at ΔV > 10 km/h [34]. Similarly, small differences in the head-to-head restraint distance in the range of 70–120 mm can increase injury risk by over 40% [35].

In rear-end collisions, the interaction between the ATD (Anthropomorphic Test Devices) and the seat is of critical importance for safety performance results. Tests are conducted with the seat in a standard position, devoid of additional accessories, and with comfort features, such as pneumatic lumbar support, disabled. Altering the seat adjustment can drastically modify the measurement outcomes; furthermore, depending on the stiffness of the seat pan and seatback, new phenomena may emerge, such as the recently identified mechanism of oscillation transfer from the seatback to the head restraint, referred to as the Pelvis-to-Headrest Transmission Effect [36].

Seat geometry and posture influence spinal alignment and neck loads. A seatback recline angle of approximately 10° and lower lumbar stiffness increase thoracic flexion by 11.4° and flatten lumbar lordosis by 8.2°, shifting the center of gravity of the head and pelvis forward by about 23 mm; a softer seat pan material with a modulus of 150–200 MPa exacerbates posterior pelvic tilt and neck loads during rapid acceleration [37]. A slumped posture is associated with a 43% increase in lumbar pressure, a 7.5° reduction in lumbar lordosis, and an 11.2° increase in cervical flexion, whereas a reclined posture increases lordosis by 9.8% and reduces seat pan pressure by 36%, thereby decreasing pelvic stability [38].

Controlled seat deformation limits injury risk, as reflected by lower NIC and Nkm values and reduced severity of the rebound phenomenon, whereas overly rigid structures increase forces in the cervical and thoracic spine and promote ramping [36]; conversely, overly soft structures delay head-to-head restraint contact and increase relative head-to-torso motion [7,10,20,39,40,41,42,43,44]. Field data indicate that vehicles with stiff seats exhibit a 25–40% higher injury risk at the same ΔV than cars with more compliant seats [41]. Newer design solutions, including perimeter frames and forward-positioned head restraints, reduce moments and head displacements but require a proper combination of stiffness and controlled deformation to avoid deteriorating risk indices [20,24].

In the literature, comfort overlays have thus far been analyzed mainly in terms of ergonomics; however, based on reports regarding seat design modifications, it can be postulated that their application will induce similar kinematic effects and translate into risk metrics. A foam stiffness range of 80–120 kPa usually provides a favorable compromise: approximately 35% less contact pressure relative to hard foams, seat pan deflection of about 15 mm (compared to about 30 mm for soft ones), a shorter stabilization time of around 140 ms, and reduced torso center of mass displacement; in simulations, this translates to lower NIC and Nkm values [5]. Deformation control is crucial because compliant, energy-absorbing seats reduce loads but may induce non-physiological motions of the lower cervical segments prior to head restraint contact; therefore, hysteresis and material creep must be limited. Layered systems prove effective, where a soft outer layer interacts with a core of higher elasticity or a gel insert, providing damping without excessive response delay [32,45]. Interaction with the seatbelt must also be considered, as increasing seat pan softness and mass may delay seatbelt engagement, modifying thoracic and pelvic kinematics [46]. In sliding or retention seats, a damping force of 3 kN for low or medium energy and approximately 6 kN for high energy is associated with a 28–34% decrease in NIC and a 31% reduction in neck bending moment without deteriorating performance in frontal collisions or rollovers [47]. Simultaneously, high-retention mechanisms and elliptical guides, by aligning the head support trajectory, limit contact delay and stabilize the occiput [48,49]. Alternative seat design modifications also include integrated solutions. The Volvo WHIPS system, in which a dual-phase seatback yield of approximately 15° reduces neck extension moments by 35–45%, head acceleration by about 30%, and axial forces by approximately 40%, as well as shortening the time to contact by 20–25 ms [50,51], requires that overlays do not disrupt the controlled seatback deformation; they should not be combined with excessive stiffening of this zone.

Seat modifications in the form of external overlays have hitherto been analyzed mainly in the context of ergonomics and comfort. However, reports regarding structural seat modifications suggest that additional foam layers of varying stiffness can alter both the initial occupant posture and the seatback deformation dynamics. Foam material parameters (80–120 kPa), delays resulting from hysteresis and creep, as well as the interaction with the seatbelt, may influence the kinematics of the chest, pelvis, and head. Simultaneously, the literature indicates a general consensus that a change in occupant posture has a direct impact on the biomechanical response and frequently leads to the emergence of new mechanisms and motion patterns. However, there is a lack of systematic studies in the literature evaluating the influence of overlays, particularly those supporting lumbar lordosis, on the biomechanical response in rear-end collisions. This constitutes a significant gap, as such overlays are commonly utilized by users yet are not accounted for in homologation tests or standard safety assessment methods. Therefore, the issue concerns not merely comfort, but the influence of lumbar overlays on the biomechanical response during a collision, an influence that has not previously been evaluated in dynamic tests involving ATD.

The objective of this study was the experimental evaluation of the influence of lumbar support seat overlays in order to capture the resulting biomechanical response under controlled dynamic conditions, utilizing standardized crash pulses and an ATD. The results will enable the determination of whether the application of lumbar overlays, despite their widespread ergonomic function, may adversely affect passive safety parameters, and consequently, whether there is a need to develop guidelines regarding their safe usage.

## 2. Materials and Methods

**Figure 1:**
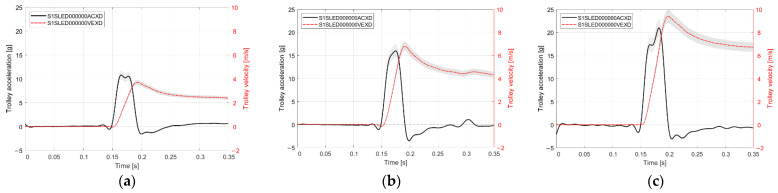
Average crash pulses: (**a**) a = 10 g, ∆v = 3.6 m/s; (**b**) a = 15 g, ∆v = 6.7 m/s; (**c**) a = 20 g, ∆v = 9.4 m/s. The gray area represents the standard deviation (SD). Labels in parentheses follow the ISO/TS 13499 naming convention.

The experiment was conducted at the Laboratory of Vehicle Dynamics and Safety, located in the GEO-3EM Research Complex (ENERGY, ECOLOGY, EDUCATION) at Wrocław University of Science and Technology. Rear impact crash pulses were reproduced using a sled crash system. An automotive seat, equipped with a compatible seatbelt assembly, was mounted on a movable trolley. The trolley was subjected to gradual acceleration, after which it impacted a barrier with energy absorbers selected to achieve the expected crash pulse. Due to the close relationship between biomechanics and injury criteria with the magnitude of the crash pulse, the range of test conditions was extended beyond standardized crash pulses, specifically the low-energy crash pulse of 10 g. The value was increased to a crash pulse of 20 g to enable a more comprehensive analysis of the investigated phenomena [17,18,20,52,53]. A crash pulse of 15 g constituted an intermediate level. The crash pulse time histories were filtered using a CFC 60 filter, in accordance with the SAE J211-1 standard. A comparative summary of the crash pulse time histories is presented in Figure 1.

**Figure 2:**
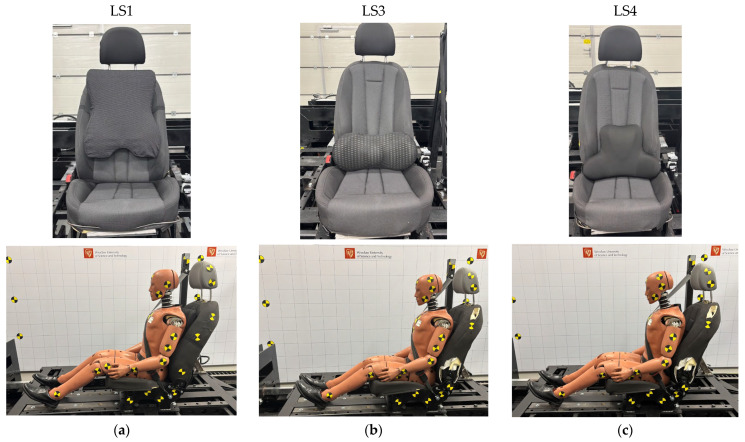
Lumbar supports (LS) used for the investigation and the dummy positioning: (**a**) lumbar supports variant 1 (LS1); (**b**) lumbar supports variant 3 (LS3); (**c**) lumbar supports variant 4 (LS4). Note that in the figure, the lumbar supports are placed on the seat solely for size perspective. During the tests, they were installed in accordance with the manufacturer’s specifications.

Reference tests were conducted with the seat positioned in the nominal setting, without the installation of additional elements. The reference signal consisted of a rear impact test with the ATD seated on the automotive seat with the seatback reclined by 21°, corresponding to the standard seating position. A seat specifically designed and constructed for the purposes of this study was utilized. The seatback was rigidly connected to the seat pan, thereby eliminating the influence of dynamic seatback deflection. Standard production components were retained, including upholstery foam and the structural frame typical of passenger vehicle seats. The ATD biomechanical response results obtained during tests with installed comfort-enhancing overlays were compared against this reference test. Three types of lumbar support (LS) overlays were selected for analysis, designated as LS1 (dimensions: 460 × 385 × 120 mm), LS3 (dimensions: 230 × 460 × 110 mm), and LS4 (dimensions: 330 × 430 × 100 mm) (Figure 2), all of which were made of memory foam and produced by the same manufacturer. It is crucial to note that the overlays in the figure are presented to provide perspective relative to the seat dimensions. During the tests, the overlays were positioned in accordance with the manufacturer’s specifications and correctly supported the lumbar lordosis. The nomenclature derives from the sequence and variants selected for analysis in this test. Comparisons were conducted relative to the reference configuration under identical crash pulses.

Safety tests utilize ATDs, which allow for the reproducible recording of loads and kinematics where research on volunteers would be unethical or impossible. Individual ATDs differ in design and mechanical response; therefore, the selection of the appropriate dummy is of critical importance for the interpretation of results: BioRID II (Biofidelic Rear Impact Dummy) most faithfully replicates head motion relative to T1, whereas the Hybrid III deviates significantly by not reproducing the early flexion phase and responding mainly with extension; furthermore, it generates higher T1 accelerations, approximately 80 m/s^2^, and three times greater extension moments than those observed in volunteers, and on a soft, production seat, it exhibits a prolonged time to head restraint contact (114–126 ms vs. ~94 ms in humans) [54,55,56]. Nevertheless, the Hybrid III dummy is utilized for injury assessment in rear impacts, and test results involving it are frequently encountered in the literature as well as in measurement protocols, for example, FMVSS 202aD [13,14,15,16]. Thus, the biomechanical response obtained via the Hybrid III dummy constitutes a useful comparative point. Additionally, BioRID, which is more suitable for investigating whiplash injuries, remains highly sensitive to changes in test configurations: installing overlays, a radically altered seat structure, or increasing the crash pulse may pose a risk of damaging the BioRID dummy or obtaining unreliable results.

The evaluation focused exclusively on linear accelerations recorded in two body segments of the dummy, the head and the chest. For each of these segments, acceleration time histories in the X, Y, and Z axes were analyzed alongside their resultant acceleration. All calculations were performed with reference to the main crash pulse time window covering the interval from 0 to 0.75 s after impact initiation.

**Figure 3:**
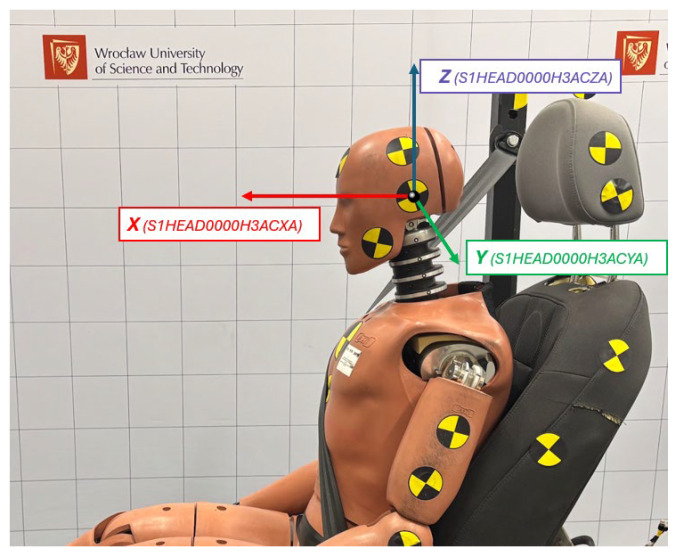
Coordinate system of the dummy’s head used in the study. Axis labels in parentheses follow the ISO/TS 13499 naming convention.

Sensor signals were recorded at a sampling rate of 15 kHz and filtered using a CFC 1000 filter in accordance with the recommendations of the SAE J211 standard. Measured data were labeled according to the ISO MME format (ISO/TS 13499). The adopted coordinate system is presented schematically in Figure 3. To verify the biomechanical response of the dummy based on sensor data, each test was additionally recorded using a Phantom VEO 410L high-speed camera operating at 3000 frames per second with a maximum resolution of 800 × 1280 pixels.

### 2.1. Analysis of Peak Acceleration Waveforms

In the first calculation stage, extreme values were determined for each acceleration waveform. This includes the maximum positive and minimum negative acceleration separately in the X, Y, and Z axes, as well as for the resultant acceleration. Extreme values were identified based on the analysis of local waveform peaks, taking into account the minimum required prominence and the minimum time interval between peaks. This was intended to eliminate random waveform fluctuations and retain only the actual peak acceleration values. For each test the maximum and minimum values along with their corresponding time instants were recorded. The collected data enabled the creation of tables containing a full set of maximum and minimum acceleration values for each body part in individual axes and for the resultant accelerations. For each crash pulse the results obtained in this manner for the LS configuration were compared with the values obtained in the reference configuration.

### 2.2. Analysis of Percentage Differences Relative to the Reference Configuration

For each crash pulse the mean peak acceleration values were calculated for the reference group, which served as the baseline. Subsequently the results obtained in the LS configuration were compared against these values. The differences were expressed as a percentage change relative to the reference group mean in accordance with Equation (1):

(1) $$\Delta \%=\frac{\left(W_{LS}-\mu_{REF}\right)}{|\mu_{REF}|}\cdot 100$$

where

$W_{LS}\;$denotes the value of the given parameter in the LS configuration,

$\mu_{REF}\;$corresponds to the mean value of this parameter in the reference group.

The analysis encompassed three primary metrics, including the maximum positive acceleration, the absolute value of the minimum negative acceleration, and the minimum negative acceleration retaining its sign. Percentage difference values were calculated for each of these metrics. Additionally, where the variance in the reference group exceeded zero, normalized values were also computed. This approach facilitated an unambiguous comparison of both configurations within individual crash pulses and ensured the interpretative consistency of the results regardless of differences between test conditions.

### 2.3. Time-Domain Waveform Analysis

In the next stage of the analysis, full acceleration waveforms were compared between the LS configuration and the reference configuration. For each crash pulse a mean reference waveform was established by averaging all measurements in the reference group for a specific body part and axis. Subsequently the LS waveforms were interpolated to a common time grid and compared with the corresponding reference waveform.

To evaluate the degree of conformity of the waveform shapes, the Root Mean Square Error (RMSE) measure was applied. Several variants of this measure were calculated. The first variant, designated as RMSE plain, defined the difference between waveforms without any time alignment. The second variant, designated as RMSE aligned, accounted for the time alignment of signals using cross-correlation, which allowed shifting one signal relative to the other within a range of small delays. The third variant, designated as RMSE scaled, was calculated after prior matching of the amplitude and signal offset so as to obtain a measure reflecting only differences in the waveform shape regardless of its magnitude. Additionally local RMSE values were calculated in short time intervals around moments corresponding to maximum and minimum values in the reference waveform, which enabled a detailed assessment of differences in the most critical moments of the crash pulse.

In the test scenarios a comparison of acceleration waveforms was conducted between the reference configuration (REF) and the LS1, LS3, and LS4 variants. Two body segments were analyzed, the head and the chest, by evaluating the differences between signals in terms of the Root Mean Square Error after time alignment (RMSE aligned) and after time and amplitude alignment (RMSE scaled). Such an approach allows distinguishing errors resulting from time offsets and intensity differences from discrepancies in the waveform shape itself.

### 2.4. CORA (CORrelation and Analysis)

An additional comparative method involved conducting an analysis modeled on the comparative analysis performed in the CORA (CORrelation and Analysis) software [57]. Its objective was to quantitatively determine the degree of similarity of full acceleration waveforms between the reference configuration and the LS configurations. The analysis was conducted for head and chest accelerations across all measurement axes, including X, Y, Z, and the resultant acceleration for all considered crash pulses. For each combination of body part axis and test scenario, the mean reference waveform was determined by averaging the signals in the reference group, whereas the LS waveforms were treated as signals compared against this baseline.

Signal conformity assessment was carried out using the CORA standard, which combines two components, the corridor rating and the cross-correlation rating. In the first step a time evaluation window covering the main interval of impact dynamics was defined for each reference waveform. Based on this the mean reference curve and its corresponding standard deviation σ(t) were determined, which served to construct inner and outer tolerance corridors around the reference waveform. The Corridor method yielding the C1_corridor score reflected the proportion of time during which the LS waveform remained within the defined corridors around the reference curve in the specified evaluation window, utilizing a continuous transition function between the region of full conformity and the region of exceedance.

In the second step the correlation component of the CORA method was applied, separating it into three independent aspects, including waveform shape conformity (V_progression), phase conformity (P_phase), and size/amplitude conformity (G_size). To this end the cross-correlation function was determined between the LS waveform and the mean reference waveform within the evaluation window, allowing for small time shifts in one signal relative to the other. Based on this the maximum value of the correlation coefficient K_max and the corresponding optimal time shift designated as time shift were determined. The V_progression variable constituted a normalized measure of shape similarity calculated as a monotonic function of K_max. The P_phase measure reflected the penalization for the time shift between signals, decreasing as the absolute value of the time shift parameter increased. In turn G_size described the conformity of the signal amplitude level based on a comparison of their energy over time.

The final evaluation of each LS configuration relative to the reference designated as C_total was determined as a weighted combination of the corridor rating C1_corridor and the cross-correlation components (V_progression, P_phase, G_size). This score was interpreted on a 0–1 scale, where values close to 1 indicated high waveform conformity, whereas values significantly below 0.6 pointed to clear discrepancies in shape, occurrence time, or the scale of the dynamic response. Additionally, values of K_max and time shift were recorded, which enabled distinguishing cases of dominant shape differences (low K_max), time shifts (large absolute value of time shift), or amplitude differences (reduced G_size with preserved shape correlation).

The CORA analysis results were compiled in the form of tables for each body part, axis, and scenario, including the values of C_total, C1_corridor, V_progression, P_phase, G_size, K_max, and time shift for the individual LS variants. Additionally, overlay plots of the LS and reference waveforms with the evaluation window marked were generated, which served for qualitative verification of the obtained numerical results and easier interpretation of the causes of low scores in selected configurations. This approach enabled a comprehensive assessment of the dynamic response conformity considering the signal’s position relative to the tolerance corridors, shape similarity, time synchronization, and conformity of the acceleration level simultaneously.

### 2.5. Data Processing

For each body segment, measurement axis, and test scenario, maximum and minimum values, their absolute values, and RMSE measures were compiled with reference to the corresponding reference waveforms. This data was recorded in the form of collective tables, which served as the basis for further comparison of the LS configurations against the reference configuration.

The objective of the described computational procedures was the quantitative determination of differences in acceleration characteristics between the two configurations. The analysis did not include clinical interpretation, and its results served as the basis for further biomechanical assessment of the LS configuration’s influence in rear-impact studies.

## 3. Results

Synthesizing the overall trends across the tested crash pulses reveals a distinct evolution in the dummy’s biomechanical response. At low-energy pulses (10 g), the primary deviation is observed in amplitude effects, where the compliance of the overlays actively dampens peak accelerations. As the energy increases to medium pulses (15 g), the system exhibits high timing sensitivity; this is the transitional phase where phase shifts are most pronounced due to the variable stabilization time of the foam. Finally, at high-energy pulses (20 g), the response shifts towards controlled deformation behavior. Under these loads, the material is fully compressed, resulting in a synchronization of waveforms and minimizing time delays, as the biomechanical response becomes dominated by the structural stiffness of the overlays rather than the initial compliance of the overlay.

### 3.1. Peak Analysis

**Figure 4:**
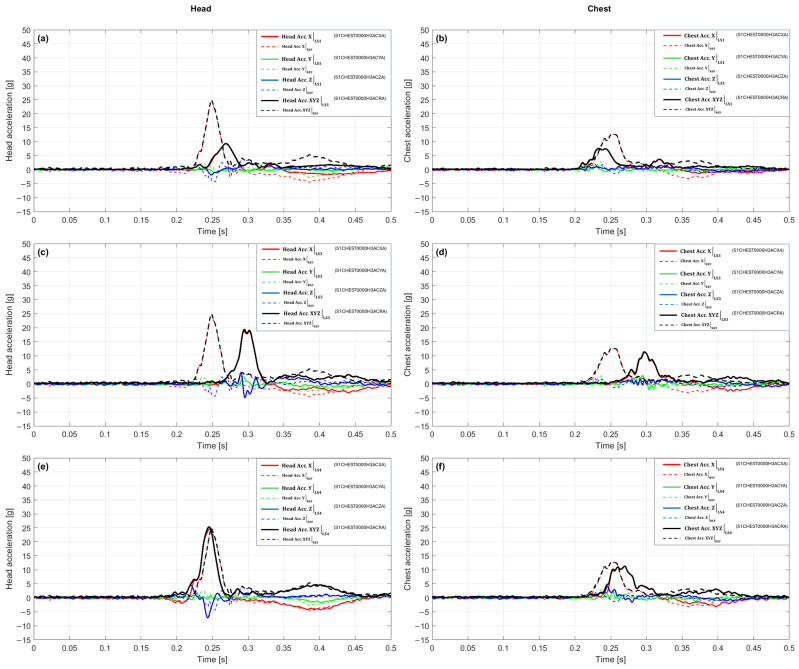
Head and chest accelerations in the X-, Y-, and Z-axes for a crash pulse of 10 g: (**a**) lumbar supports variant 1 (LS1)—head; (**b**) lumbar supports variant 1 (LS1)—chest; (**c**) lumbar supports variant 3 (LS3)—head; (**d**) lumbar supports variant 3 (LS3)—chest; (**e**) lumbar supports variant 4 (LS4)—head; (**f**) lumbar supports variant 4 (LS4)—chest. Dashed lines indicate the reference configuration (REF), while solid lines indicate the LS variants. Labels in parentheses follow the ISO/TS 13499 naming convention.

In the scenario with a 10 g crash pulse, waveforms from the LS1 variant were compared with the reference configuration (REF), referencing Figure 4a,b. In the head (Figure 4a) the first maximum in the reference configuration was 24.88 g at 0.249 s, while the LS1 variant recorded 9.34 g at 0.269 s. This signifies an amplitude reduction of 62.5% and a delay in the time of maximum of 0.020 s. The plot shows that the culmination for LS1 appears later and is distinctly lower; the signal rise to the peak is slower than in the reference configuration, and a gentler decay is observed after the culmination. The peak value remains dominated by the *X*-axis component, while the Y and Z components do not reach comparable values at the same moment, which limits the resultant acceleration magnitude. Such a waveform indicates that in the head, the action of LS1 translates into a reduction in the peak load and an extension of the time to maximum, resulting in a slower and milder dynamic response.

In the chest (Figure 4b) the first maximum in the reference configuration was 12.71 g at 0.254 s, while the LS1 variant achieved 7.55 g at 0.234 s. This corresponds to an amplitude drop of 40.6% and an acceleration of the time of maximum of 0.020 s. The plot shows that the peaks for LS1 appear earlier and at a lower level, suggesting a faster yet less intense response. Similarly to the head, the acceleration culmination coincides with the maximum of the X-component, whereas the lack of co-occurring extremes in the Y and Z axes limits the resultant value. Consequently, the action of LS1 in the chest leads to a significant dampening of the amplitude and a shortening of the response rise time relative to the reference configuration. The plot shows that the peaks for LS1 appear slightly earlier and at a lower level, indicating a faster but less intense dynamic response buildup. Similarly to the head, the acceleration culmination remains dominated by the X-component, whereas the lack of co-occurring extremes in the Y and Z axes limits the total resultant value. Consequently, the action of LS1 in the chest leads to a significant dampening of the amplitude and a shortening of the response rise time relative to the reference configuration.

Figure 4c,d presents a comparison of the dynamic responses for the LS3 variant and the reference configuration. In the head (Figure 4c), a clear waveform phase shift is visible, where in the reference system the maximum is reached earlier (24.88 g at 0.249 s), while in the LS3 variant the peak only appears after 0.297 s, with a smaller amplitude of 19.37 g. This difference corresponds to a decrease of about 22% and indicates that the action of LS3 results in a slowing down and weakening of the head system’s response. In contrast to the reference waveform, where the acceleration rises sharply, the LS3 signal increases gradually, and the culmination is less distinct. The acceleration maximum is primarily associated with the dominant *X*-axis component, while the Y and Z components remain relatively low at that moment, which limits the resultant value. Overall, the LS3 waveform features a milder time profile, more distributed in the rising phase, suggesting that the system reacts more slowly but with a lower peak load. Figure 4d shows a clear difference in the acceleration waveforms of the chest between the reference configuration and the LS3 variant. In the case of the reference system, the maximum reaches 12.71 g at 0.254 s, whereas in the LS3 configuration, the peak shifts to 0.297 s and reaches 11.47 g. This signifies an amplitude reduction of 9.8%, combined with a distinct delay in the moment of culmination by 0.043 s. The shape of the LS3 curve suggests that the chest response develops more slowly, and the signal rises over a longer time interval, while reaching the maximum value is preceded by a phase of gentle increase. After the culmination, no sudden drop is observed, but a smooth dampening of the acceleration, which indicates a more temporally distributed system response. Compared to the reference configuration, the behavior of LS3 can be described as less dynamic but more controlled, with limited acceleration jumps and an extended reaction time to the crash pulse.

Figure 4e,f presents the acceleration waveforms for the LS4 variant compared to the reference configuration. In the case of the head (Figure 4e), the first maximum in the reference configuration was 24.88 g at 0.249 s, whereas in the LS4 variant, 25.34 g was recorded at 0.245 s. This corresponds to a slight amplitude difference (an increase of 1.8%) and an acceleration of the moment of maximum by 0.004 s. The shape of the LS4 waveform remains very close to the reference one, and the rising phase is almost identical; however, the culmination occurs slightly earlier and at a minimally higher peak value. After reaching the maximum, the LS4 curve drops faster, which indicates a shorter duration of the intensive reaction phase. At the moment of culmination, the *X*-axis component has the main contribution to the total value, while the remaining axes do not additionally amplify the signal, which limits further increase in the acceleration value. Figure 4f shows a large convergence of the acceleration waveforms for the LS4 variant and the reference configuration, although subtle differences in the reaction dynamics are noticeable. In the chest, the maximum acceleration in LS4 is 11.32 g at 0.270 s, while in the reference configuration, it reached 12.71 g at 0.254 s. This signifies an amplitude reduction of 10.9% and a delay in the time of culmination by 0.016 s. The LS4 signal rise proceeds similarly to the baseline configuration; however, the maximum occurs slightly later, and the peak shape is more rounded and less distinct. After the culmination, the acceleration decreases faster, and the subsequent portion of the curve has a milder course, which shortens the duration of the intensive reaction phase. Overall, the LS4 signal in the chest is characterized by a lower abruptness of changes and a more subdued dynamic profile, suggesting a limitation of the load in the final phase of the crash.

The integrated view of the scenario with a 10 g crash pulse, compiled in Table 1, confirms the dominant effectiveness of the LS1 variant, which shows clear amplitude dampening in both analyzed segments. In the head, LS1 reduces the peak value relative to the baseline by 62.46% and simultaneously delays the time of maximum by 20 ms, which is reflected by the indicators “Δ% Head vs. REF” and “Δt Head [ms]”. The LS3 variant reduces the amplitude by 22.16%, with a peak delay of 48 ms, while LS4 maintains the resultant value slightly above the baseline (+1.84%) and accelerates the maximum by 4 ms. In the chest, all variants lead to an amplitude reduction. LS1 provides the deepest dampening (−40.63%) and earlier peak occurrence (−20 ms), while LS3 and LS4 act milder (−9.77% and −10.97%), and their maxima are time-shifted towards the end of the pulse. Overall, the compilation confirms that LS1 represents the most protective response character, LS3 is an intermediate system with moderate dampening and delayed reaction, while LS4 exhibits an atypical combination of no dampening in the head and moderate dampening in the chest, with a slight acceleration of the peak in the head part.

**Figure 5:**
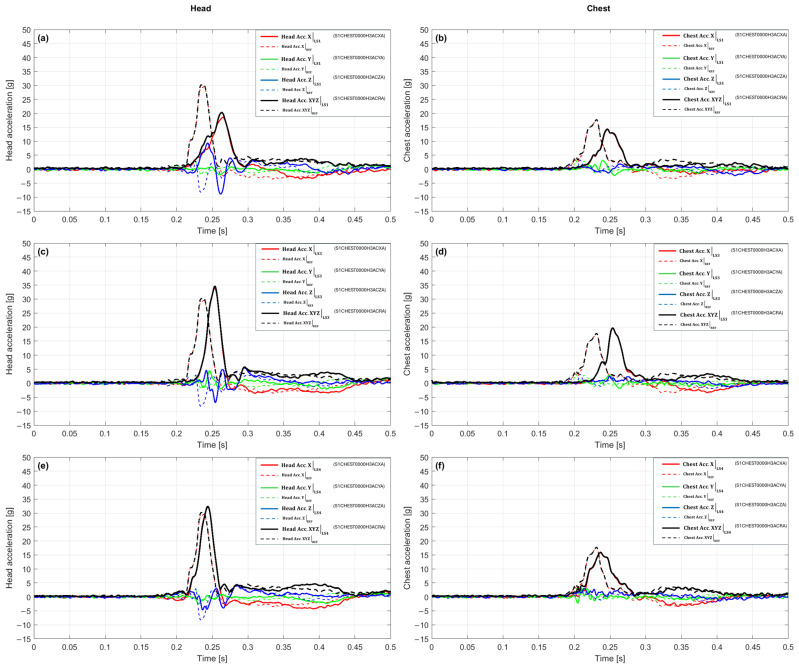
Head and chest accelerations in the X-, Y-, and Z-axes for a crash pulse of 15 g: (**a**) lumbar supports variant 1 (LS1)—head; (**b**) lumbar supports variant 1 (LS1)—chest; (**c**) lumbar supports variant 3 (LS3)—head; (**d**) lumbar supports variant 3 (LS3)—chest; (**e**) lumbar supports variant 4 (LS4)—head; (**f**) lumbar supports variant 4 (LS4)—chest. Dashed lines indicate the reference configuration (REF), while solid lines indicate the LS variants. Labels in parentheses follow the ISO/TS 13499 naming convention.

Figure 5a,b presents a comparison of the acceleration waveforms for the LS1 variant and the reference configuration for the 15 g crash pulse. In the head (Figure 5a), the maximum value in the reference configuration was 30.41 g at 0.238 s, while 20.27 g was recorded in LS1 at 0.263 s. This signifies an amplitude reduction of about 33% and a shift in the time of culmination towards later occurrence by 0.025 s. The shape of the LS1 waveform remains similar to the reference one, but the dynamics of the rise are distinctly milder, and the peak is reached more slowly and at a lower level. After culmination, the signal damps smoothly, without secondary fluctuations. The resultant acceleration value is shaped mainly by the *X*-axis component, while the remaining axes do not reach comparable values at the same time. As a result, a reduction in maximum head acceleration and an extended rise time are observed, which indicates more effective pulse dampening in this configuration. In the chest (Figure 5b), the maximum in the reference configuration was 17.75 g at 0.231 s, and in the LS1 variant, it reached 14.37 g at 0.246 s, which corresponds to an amplitude drop of 19% and a delay in the time of maximum by 0.015 s. In the initial rising phase, the curves are similar; however, in LS1, the signal rises more slowly, and the peak occurs later and at a lower level. After reaching the maximum, the LS1 waveform maintains a gentle character, and the rate of decay remains close to the reference. The largest contribution to the resultant value is again from the *X*-axis component, while the transverse components remain relatively small. Overall, LS1 leads to a reduction in peak values and a slowing of the chest reaction dynamics, which confirms its dampening action in the analyzed scenario.

With a 15 g crash pulse, the waveforms for the LS3 variant (Figure 5c,d) exhibit a distinct shape compared to the reference configuration, both in terms of amplitude and the time of maximum occurrence. In the head (Figure 5c), a maximum of 34.57 g was recorded at 0.254 s, which corresponds to an amplitude increase of about 14% and a delay in the time of culmination by 0.016 s relative to the baseline. The LS3 variant is characterized by a longer rising phase and a distinct, narrow peak, after which the acceleration rapidly decreases. In the final phase, no additional local maxima occur, and the shape of the waveform remains symmetrical with respect to the culmination. The resultant value at the time of maximum is primarily determined by the *X*-axis component, while the components in the Y and Z axes maintain a lower level, which limits their influence on the total acceleration. In the chest (Figure 5d), the maximum was 19.65 g at 0.254 s, which constitutes an amplitude increase of about 11% and a delay in the time of maximum by 0.023 s. The LS3 waveform shows a faster rise than in LS1, and the culmination is more distinct and shorter in duration. After the peak, a gradual decrease in acceleration is observed without secondary oscillations. The change in the curve shape is similar in both analyzed segments, while maintaining a comparable temporal sequence of signal rise and decay.

In the scenario with a 15g crash pulse, the waveforms for the LS4 variant (Figure 5e,f) exhibit different behaviors in the head and chest segments. In the head (Figure 5e), a maximum of 32.39 g was recorded at 0.244 s, which signifies a slight amplitude increase of about 6.5% and a delay in the time of culmination by 0.006 s relative to the reference configuration. The LS4 waveform retains the overall shape of the reference curve, but the rising phase is slightly prolonged, and the acceleration peak occurs slightly later and reaches a higher value. After culmination, a gentle decrease in acceleration is observed without additional local extrema, and the decay profile remains similar to the baseline. The resultant value at the time of maximum continues to be shaped mainly by the *X*-axis component, while the components in the Y and Z axes remain at a lower level. In the chest (Figure 5f), the maximum reached 15.93 g at 0.237 s, which corresponds to an amplitude drop of about 10% and a delay in the time of culmination by 0.006 s relative to the baseline. The LS4 waveform is characterized by a similar beginning of the rise; however, the culmination occurs later and at a lower level. After the maximum, the acceleration decreases smoothly, without secondary amplitude changes. The entire signal maintains a regular shape, and the differences between LS4 and the reference concern mainly the height and time of peak occurrence. The LS4 variant thus maintains a similar temporal course in both analyzed segments, with a slight increase in the peak value in the head and an amplitude reduction in the chest, which confirms the stable and repeatable nature of the response.

The integrated view for the 15 g crash pulse, compiled in Table 2 for this scenario, shows a clear amplitude and time differentiation between the LS variants. In the head, the reference value is 30.41 g at 0.238 s. The LS1 variant achieves 20.27 g, which signifies an amplitude drop of 33.3%, and the maximum occurs at 0.263 s, 0.025 s later. The LS3 variant yields 34.57 g, representing an increase of 13.7%, with culmination at 0.254 s (+0.016 s relative to the baseline). The LS4 variant achieves 32.39 g, which is 6.5% more than the reference, and the maximum occurs at 0.244 s, signifying a delay of 0.006 s. In the chest, the baseline value is 17.75 g at 0.231 s. The LS1 variant achieves 14.37 g (−19.1%) at 0.246 s (+0.015 s), while LS3 yields 19.65 g (+10.7%) with a maximum at 0.254 s (+0.023 s). The LS4 variant gives 15.93 g, which means a drop of 10.3% and the occurrence of the maximum at 0.237 s (+0.006 s). The compilation of these values in the columns Peak [g], Δ% vs. REF, and t_peak [s] shows that in the head, only LS1 leads to a clear reduction in the peak value, while LS3 and LS4 cause an increase with a slight delay in the time of maximum. In the chest, the differences are more varied, i.e., LS1 and LS4 reduce the amplitude, while LS3 shows a moderate increase, and all variants are characterized by a slight shift in the maximum towards later times. Due to the lack of reliable data for the pelvis, values for this region are not included in the compilation.

**Figure 6:**
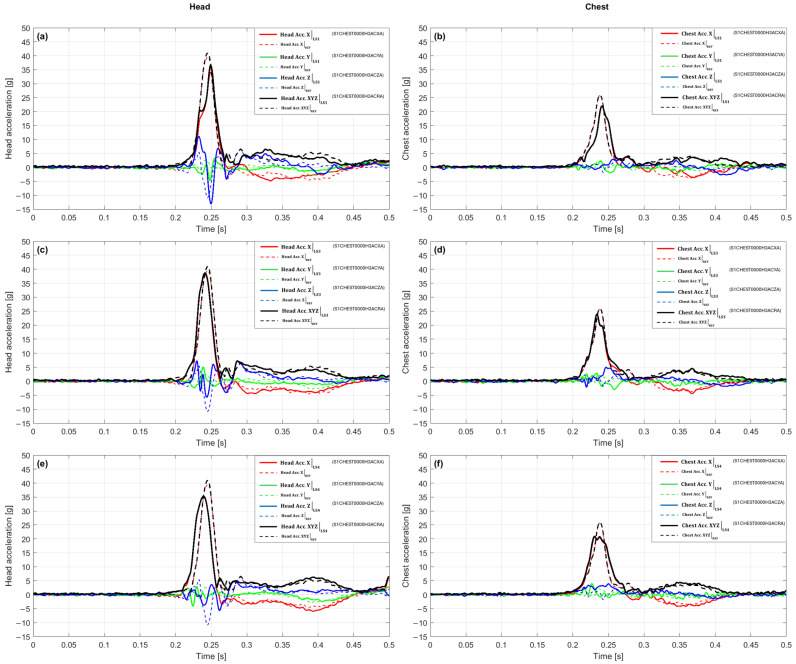
Head and chest accelerations in the X-, Y-, and Z-axes for a crash pulse of 20 g: (**a**) lumbar supports variant 1 (LS1)—head; (**b**) lumbar supports variant 1 (LS1)—chest; (**c**) lumbar supports variant 3 (LS3)—head; (**d**) lumbar supports variant 3 (LS3)—chest; (**e**) lumbar supports variant 4 (LS4)—head; (**f**) lumbar supports variant 4 (LS4)—chest. Dashed lines indicate the reference configuration (REF), while solid lines indicate the LS variants. Labels in parentheses follow the ISO/TS 13499 naming convention.

In the 20 g crash pulse scenario, the reference configuration achieved a maximum value of 40.98 g at 0.245 s in the head, and 25.88 g at 0.238 s in the chest. Against this background, the LS1 variant waveforms (Figure 6a,b) show a reduction in peak values and slight temporal shifts, while maintaining a similar general shape of the response. In the head (Figure 6a), a maximum of 36.67 g was recorded at 0.249 s, which corresponds to an amplitude drop of 10.5% and a delay in time of culmination by 0.004 s relative to the baseline. The signal rise begins at a similar time; however, in LS1, it has a milder character, the pre-peak slope is less steep, and the transition to maximum is smoother. The culmination occurs slightly later and is characterized by a lower peak value, while after its occurrence, a clear signal dampening is observed without secondary extrema. In the acceleration vector structure, the *X*-axis component plays a dominant role, accounting for the main direction of the pulse, while the Y and Z components retain smaller values and do not significantly affect the total maximum. In the chest (Figure 6b), the maximum was reached at 21.99 g at 0.241 s, which constitutes a reduction of about 15% and a delay of 0.003 s relative to the baseline. In the rising segment, both configurations show a similar course, but in LS1, the value increase process is more temporally distributed, resulting in a milder, less abrupt peak. After reaching the maximum, the signal decays smoothly and stably, without additional local extrema. The LS1 waveform maintains phase consistency with the reference, and both the time of rise initiation and decay occur at the same time points.

In the 20 g crash pulse scenario, the waveforms for the LS3 variant (Figure 6c,d) indicate a reduction in acceleration peak values and an earlier occurrence of the time of culmination compared to the baseline configuration. In the head (Figure 6c), a maximum of 38.66 g was recorded at 0.241 s, which corresponds to an amplitude drop of 5.7% and an acceleration of the time of maximum by 0.004 s. The LS3 waveform maintains the general shape of the pulse; however, differences appear in the final rising phase, the values increase slightly slower, and the peak is reached earlier and at a lower value than in the reference configuration. The culmination phase is shorter and more regular, and after its completion, the acceleration decreases smoothly, without secondary extrema. In the acceleration vector structure, the *X*-axis component plays a dominant role, while the Y and Z components retain smaller values and do not increase the resultant amplitude. In the chest (Figure 6d), the maximum was 23.95 g at 0.234 s, which signifies an amplitude reduction of 7.5% and an acceleration of the time of culmination by 0.004 s. The initial LS3 waveform is consistent with the reference configuration; however, in the final rising phase, there is an earlier achievement of a smaller peak value. The pre-peak slope has a similar inclination, while after the maximum, the signal decreases faster and stably, without secondary amplitude changes. At the time of culmination, the *X*-axis component remains the dominant element of the acceleration vector, and the other directions show no significant deviations.

In the 20 g crash pulse scenario, the waveforms for the LS4 variant (Figure 6e,f) show a clear reduction in peak values and a shift in the time of culmination relative to the baseline configuration. In the head (Figure 6e), the maximum reached 35.26 g at 0.239 s, which corresponds to an amplitude drop of 13.97% and an advancement of the time of maximum by 0.006 s. In the initial phase, the LS4 waveform remains similar to the reference configuration; however, differences become apparent in the final rising segment, the acceleration value increases more slowly, and the peak is reached earlier and at a lower level. The culmination phase is shorter and more symmetrical, and after its completion, the acceleration decreases uniformly, without additional local extrema. The *X*-axis component retains its dominant influence on the resultant value, while the Y and Z components maintain lower values and do not cause an increase in the total amplitude. In the chest (Figure 6f), a maximum of 20.89 g was recorded at 0.231 s, which signifies an amplitude reduction of 19.27% and an advancement of the time of culmination by 0.007 s. The shape of the LS4 waveform in the initial phase is consistent with the baseline; however, in the final rising part, an earlier achievement of the maximum value and a lower peak level are observed. After culmination, the signal drops stably, and its temporal course remains regular and devoid of secondary fluctuations. The changes are quantitative: they do not affect the general structure of the waveform, but they clearly indicate a limitation of the maximum acceleration values relative to the baseline configuration.

The summary of the results for the 20 g crash pulse scenario, which presents a complete picture of the amplitude and temporal changes between the baseline configuration and the LS1, LS3, and LS4 variants, is shown in Table 3. The reference values for this scenario are 40.98 g at 0.245 s in the head and 25.88 g at 0.238 s in the chest. In the head, the LS1 configuration achieved a maximum of 36.67 g at 0.249 s, which represents a drop of 10.53% and a delay in the time of culmination by 0.004 s. The LS3 variant shows a maximum of 38.66 g at 0.241 s, meaning a drop of 5.67% and an advancement of the peak by 0.004 s, while LS4 recorded 35.26 g at 0.239 s, which signifies an amplitude reduction of 13.97% and an advancement of the time of maximum by 0.006 s. As a result, all LS variants lead to an amplitude reduction while maintaining a similar waveform shape and slight temporal differences. In the chest, the reference value was 25.88 g at 0.238 s. The LS1 variant reached 21.99 g at 0.241 s, which corresponds to a reduction of 15.00% and a delay of 0.003 s. For LS3, the maximum was 23.95 g at 0.234 s, meaning a drop of 7.46% and an advancement of 0.004 s, while LS4 yielded 20.89 g at 0.231 s, which corresponds to a reduction of 19.27% and an advancement of 0.007 s. These values indicate a consistent amplitude reduction in the chest for all variants, with differences in peak chronology not exceeding a few milliseconds. The compilation of results in the columns Peak [g], Δ% vs. baseline, and t_peak [s] confirms the consistency of the trends between the analyzed segments. In the head and chest, the LS variants lead to a reduction in maximum values ranging from 5% to 19%, while maintaining a similar temporal course. The differences primarily concern amplitude, while changes in the time of peak occurrence range from −0.007 to +0.004 s, which indicates the maintenance of a uniform temporal characteristic throughout the entire system.

### 3.2. RMSE Analysis

For the 10 g crash pulse in the head, the RMSE aligned values ranged from 0.79 g for LS1 to 0.91 g for LS3, with an intermediate value of 0.85 g for LS4 (Table 4). After amplitude matching (which involved proportionally strengthening or weakening the LS signal so that its range of values corresponded to the reference signal), the errors decreased to 0.54 g (LS1), 0.63 g (LS3), and 0.59 g (LS4). The error reduction of about 30% indicates that the main differences between LS and the baseline resulted from the LS sensors recording the same waveform but with a slightly lower intensity.

The LS1 variant achieved the highest agreement with the reference waveform, both before and after amplitude matching. This means that LS1 faithfully reproduced the temporal structure of the signal, and the differences resulted primarily from the level of recorded acceleration. The LS3 variant maintained a similar signal shape, but its amplitude was further removed from the baseline. LS4 held an intermediate position: the waveform was well matched in time, but it still exhibited a lower intensity of accelerations relative to the reference configuration.

For the chest, the RMSE aligned errors were higher and averaged 1.02 g for LS1, 1.10 g for LS3, and 1.05 g for LS4. After amplitude matching, they dropped to 0.74 g, 0.81 g, and 0.78 g, respectively. This trend is consistent with the observations for the head; LS1 maintained the best fit, LS3 was characterized by a larger error, and LS4 ranked in between them. This means that LS1 most faithfully reproduced both the shape and the rate of acceleration changes, LS3 recorded a signal with greater dispersion and amplitude differences, and LS4 maintained correct chronology but with less precision in intensity mapping.

The average temporal shifts between the LS signals and the reference amounted to about 9–11 ms. The largest delay occurred for LS3, and the smallest for LS1. The adopted evaluation threshold was ±8 ms; thus, in some tests, the temporal differences were slightly larger. From the perspective of dynamic measurements, however, these values fall within the range considered acceptable, in accordance with the guidelines of SAE J211/1 (Instrumentation for Impact Test—Part 1: Electronic Instrumentation) and ISO 6487:2022 (Road vehicles—Measurement techniques in impact tests—Instrumentation), which specify acceptable temporal differences at the level of ±10 ms.

A comparison of the RMSE aligned and RMSE scaled values shows that amplitude matching reduced the error by 25–30%, which means the dominant source of differences between LS and REF was lower signal intensity, and not a different waveform shape (Table 4). The LS1 variant achieved the smallest total error (0.54 g after matching), which demonstrates the best agreement with the baseline. LS3 was characterized by the largest error (0.63 g) and the largest delay, while LS4 held an intermediate position; the errors were moderate, and temporal synchronization remained correct.

In the 15 g crash pulse scenario, for the head, the RMSE aligned values were: 0.83 g for LS1, 1.04 g for LS3, and 0.91 g for LS4 (Table 5). After introducing amplitude matching, meaning proportional strengthening or weakening of the LS signal so that its value range overlapped with the reference signal, the error was reduced to 0.58 g (LS1), 0.72 g (LS3), and 0.63 g (LS4). The error reduction of about 25–30% indicates that the main cause of the differences between LS and REF was the varying intensity of recorded accelerations, and not the distortion of the waveform shape. Among the analyzed variants, LS1 most accurately reproduced the reference signal, LS3 showed greater amplitude deviations and less stable mapping in time, while LS4 held an intermediate position; the shape and phase of the signal were well matched, though the amplitude remained somewhat lower than in the reference configuration.

In the case of the chest, the RMSE aligned values were higher and averaged 1.15 g for LS1, 1.26 g for LS3, and 1.19 g for LS4. After applying amplitude matching, the error dropped to 0.83 g (LS1), 0.95 g (LS3), and 0.88 g (LS4). The same pattern held as for the head: LS1 provided the best fit, LS3 generated the largest deviations, while LS4 ranked in between them. This means that the LS1 variant most faithfully reproduced both the waveform shape and the dynamics of acceleration changes, LS3 recorded a more dispersed signal burdened with larger amplitude differences, and LS4 correctly mapped the chronology of events, but with moderately reduced intensity relative to the baseline.

The compilation of RMSE aligned and RMSE scaled shows that amplitude matching allowed for an error reduction of 25–30%, which confirms that the key source of divergence between LS and REF was the differences in signal strength, and not its shape (Table 5). The LS1 variant achieved the most favorable parameters, the smallest total error after scaling (0.58 g) and the most stable temporal mapping. In contrast, LS3 was characterized by the highest error values (0.72 g) and local temporal shifts, while LS4 maintained a correct waveform shape, but with moderately reduced amplitude.

In the 20 g crash pulse scenario, for the head, the obtained RMSE aligned values were 0.92 g for the LS1 configuration, 1.18 g for LS3, and 1.01 g for LS4 (Table 6). After applying amplitude matching (meaning linear scaling of the LS signal so that its range overlapped with the range of the reference signal), the error values decreased to 0.68 g (LS1), 0.84 g (LS3), and 0.73 g (LS4). The drop of the order of 25–30% indicates that the essential difference between the LS and REF signals resulted from the level of acceleration amplitude, and not from significant distortions of their waveform shape. LS1 proved to be the variant with the smallest error and highest convergence with the baseline waveform; LS3 was characterized by the largest deviations both in terms of amplitude and temporal mapping, while LS4 ranked in between them, ensuring correct temporal matching with a slightly weaker signal intensity.

For the chest, the RMSE aligned values were generally higher and amounted to 1.22 g (LS1), 1.34 g (LS3), and 1.28 g (LS4). After introducing amplitude matching, an error reduction was recorded to 0.90 g for LS1, 1.03 g for LS3, and 0.95 g for LS4. The pattern of dependencies remained similar to that observed for the head: LS1 consistently generated the lowest error values, LS3 was characterized by the most dispersed signal and larger response delays, while LS4 maintained intermediate results. In practice, this means that LS1 most faithfully reproduced the reference signal in terms of both shape and the rate of acceleration changes, LS3 showed the largest amplitude and chronology discrepancies, and LS4 maintained a correct waveform with somewhat reduced intensity.

A comparison of RMSE aligned with RMSE scaled shows that the application of amplitude scaling allowed for an error reduction of about 25–30%, which confirms that the discrepancies between LS and REF were primarily characterized by differences in signal strength, and not in its shape (Table 6). The LS1 variant achieved the lowest total error after scaling (0.68 g) and the most stable temporal mapping. The LS3 configuration remained burdened with the largest deviations (0.84 g) and local temporal shifts, while LS4 was characterized by a moderate error level (0.73 g) while maintaining correct synchronization with the reference signal.

### 3.3. CORA Waveform Analysis

**Figure 7:**
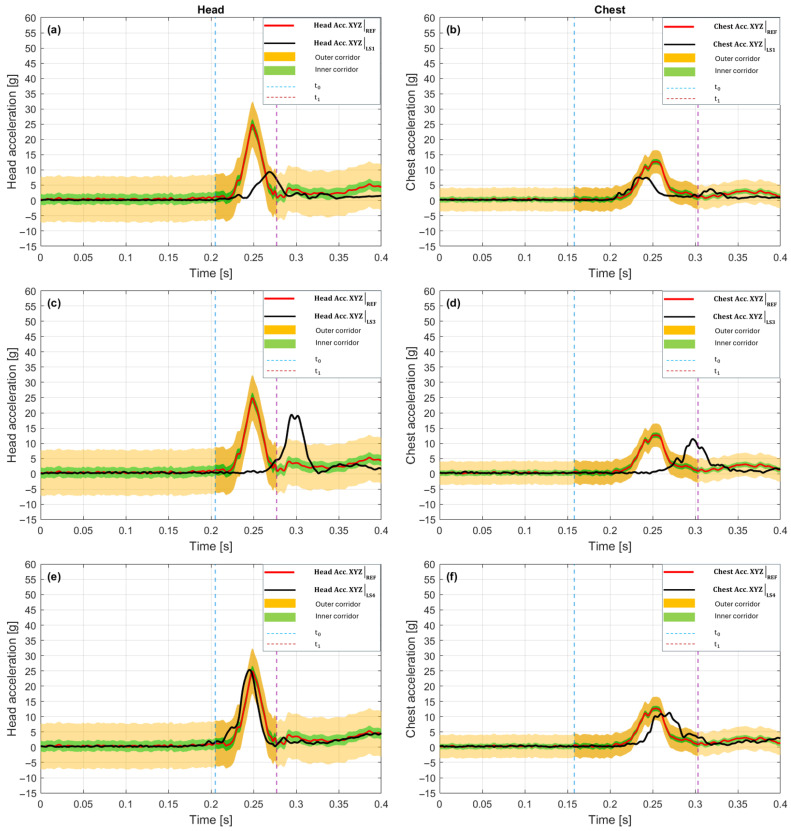
Correlation analysis of head and chest resultant accelerations for a crash pulse of 10 g: (**a**) lumbar supports variant 1 (LS1)—head; (**b**) lumbar supports variant 1 (LS1)—chest; (**c**) lumbar supports variant 3 (LS3)—head; (**d**) lumbar supports variant 3 (LS3)—chest; (**e**) lumbar supports variant 4 (LS4)—head; (**f**) lumbar supports variant 4 (LS4)—chest. The red line represents the reference signal (REF), while the black line represents the LS variant. The inner (green) and outer (yellow) corridors indicate tolerance limits. Vertical dashed lines mark the evaluation time interval [t_0_, t_1_].

In the 10 g crash pulse scenario, the LS1 variant (Figure 7a—Head, Figure 7b—Chest) showed pronounced temporal shifts and amplitude differences relative to the baseline, despite very high waveform shape match in both analyzed areas. In the head, the time shift = +18 ms value signifies a delay relative to the reference waveform by 18 ms, and P_phase = 0.000 confirms a lack of phase match in the analysis range [t_0_, t_1_].

Figure 7a shows that the LS1 peak occurred later than the maximum of the reference signal (red line), and the waveform remained outside the tolerance corridor for most of the time, which is reflected in the low C1_corridor value of 0.422. Additionally, G_size = 0.176 means that the total energy of the LS1 signal in the head is lower by as much as 82.4% relative to the baseline. Despite this, V_progression = 0.993 and Kmax = 0.987 confirm a very high curve shape match.

In the chest (Figure 7b), an inverse shift character is indicated; in this case, a time shift = −12 ms means that the LS1 signal maximum occurred 12 ms earlier than in the reference waveform. This value is still significant, but the influence on the phase metric is smaller, P_phase = 0.172, which signifies partial temporal match. The tolerance corridor coverage is better than in the head, C1_corridor = 0.679, confirming a larger part of the signal remains within the outer corridor. The signal amplitude in the chest remained reduced, G_size = 0.347, which signifies an energy drop of over 65% relative to the baseline.

The shape maintains a very good match (V_progression = 0.996, Kmax = 0.991). The overall scores C_total for both areas are similar, 0.406 (Head) and 0.592 (Chest), indicating a relatively uniform, though low level of match.

A common element in the behavior of the LS1 system in both areas is the consistent waveform profile (V_progression > 0.99), while the main sources of mismatch remain amplitude differences and temporal shifts, which have opposite signs—a delay in the head (+18 ms) and an advancement in the chest (–12 ms).

The LS3 variant shown in Figure 7c for the head and Figure 7d for the chest exhibited the most pronounced temporal delays in the entire group. In the head, the time shift = +42 ms signifies that characteristic waveform features (e.g., maximum acceleration) occurred 42 ms later than in the reference waveform. Such a large delay results in a P_phase value = 0.000, which by CORA definition means total lack of temporal match in the designated window [t_0_, t_1_] [57]. Despite this, the shape match remains high (V_progression = 0.994, Kmax = 0.989), indicating that the signal structure was well mapped, but shifted in time. The G_size value = 0.381 means that the total signal energy is lower by 61.9% relative to the baseline. Figure 7c confirms this assessment: the LS3 curve (black) rises similarly to the reference, but reaches its peak with a large delay, outside the central analysis zone. A significant part of the waveform is outside the tolerance corridor, resulting in C1_corridor = 0.467. The overall match score C_total = 0.463 is lower than in LS1, which primarily results from the larger phase shift. In the chest, the delay is more pronounced. Time shift = +53 ms, which again translates to P_phase = 0.000. The maximum acceleration in LS3 occurred 53 ms after the reference peak moment. On Figure 7d, this phase drift was visible as the main peak shifted toward the end of the time window. Tolerance corridor coverage was weaker than in the head. C1_corridor = 0.508. Despite the shift, the waveform shape remains coherent (V_progression = 0.992, Kmax = 0.983), and the amplitude reaches the highest level among the analyzed variants. G_size = 0.927, meaning only 7.3% lower than the baseline. The overall score C_total = 0.574 is slightly higher than in the head, but still remains in the range of an incomplete match.

The LS3 performance is characterized by high shape and amplitude coherence, but its main limitation in both areas is the significant temporal shift. High time shift values in the head (+42 ms) and the chest (+53 ms) suggest a global delay in the reaction of the LS3 measurement system.

The LS4 variant (Figure 7e,f) presents the best coherence with the baseline in both areas. In the head, the temporal difference relative to the reference was time shift = −5 ms, meaning the LS4 signal maximum occurred 5 ms earlier than in the reference waveform. This value falls within the typical error tolerance and results in P_phase = 0.306, which signifies partial phase match. Figure 7e shows that the LS4 peak is very close to the reference peak, and a significant part of the waveform is within the outer corridor, with fragments covering the inner corridor. This is also confirmed by the metrics, C1_corridor = 0.505, V_progression = 0.997, G_size = 0.913, Kmax = 0.994. The overall score C_total = 0.622 is the highest among all analyzed configurations in the head. In the chest, a positive temporal shift was observed: time shift = +15 ms, which translates to P_phase = 0.000. This result is lower than in the head. The main maximum of the LS4 signal occurs here, 15 ms after the baseline, which indicates nonuniform dynamics of pulse propagation along the body. Figure 7f shows that although the signal shape is well mapped (V_progression = 0.994, Kmax = 0.989), and the amplitude reaches 87.6% of the baseline value (G_size = 0.876), the phase differences cause the overall score to drop to C_total = 0.595. Tolerance corridor coverage is comparable to the head (C1_corridor = 0.567).

The LS4 variant features the most balanced match profile (both in relation to shape and amplitude) and relatively low temporal shifts. There is a difference in the sign of the time shift between the head (–5 ms) and the chest (+15 ms).

**Figure 8:**
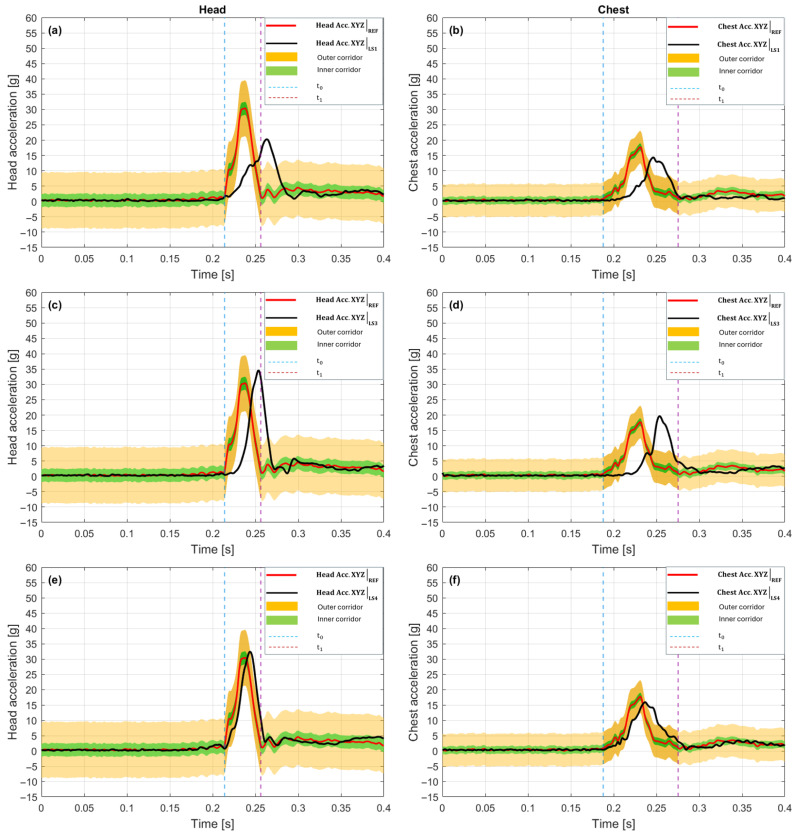
Correlation analysis of head and chest resultant accelerations for a crash pulse of 15 g: (**a**) lumbar supports variant 1 (LS1)—head; (**b**) lumbar supports variant 1 (LS1)—chest; (**c**) lumbar supports variant 3 (LS3)—head; (**d**) lumbar supports variant 3 (LS3)—chest; (**e**) lumbar supports variant 4 (LS4)—head; (**f**) lumbar supports variant 4 (LS4)—chest. The red line represents the reference signal (REF), while the black line represents the LS variant. The inner (green) and outer (yellow) corridors indicate tolerance limits. Vertical dashed lines mark the evaluation time interval [t_0_, t_1_].

The LS1 variant in the 15 g crash pulse (Figure 8a—Head, Figure 8b—Chest) was characterized by reduced match quality in terms of both amplitude and phase match, despite high signal shape correlation. In the head, the temporal shift value time shift = +12 ms indicates that the maximum of the analyzed signal occurs 12 ms after the peak in the reference waveform. Such a difference causes the P_phase = 0.000, which, according to the CORA assessment standard, corresponds to total lack of temporal match in the analyzed window [57]. For the head Figure 8a, the value C1_corridor = 0.168 indicates very limited tolerance corridor coverage. A significant portion of the LS1 signal in the head lies outside the permissible range relative to the baseline. The observed discrepancies are amplified by the low G_size value = 0.228, which means a drop in total dynamic energy of over 77% relative to the reference waveform. Nevertheless, the curve shape match is still very high (V_progression = 0.991, Kmax = 0.982), meaning that the LS1 signal in the head retains a structure similar to the baseline, but is weaker and temporally shifted. Consequently, the overall match quality score (C_total = 0.287) is low. In the chest (Figure 8b), an even larger temporal shift was recorded: Time shift = +23 ms, which again translates to P_phase = 0.000. Figure 8b indicates that the LS1 signal maximum occurs noticeably after the reference signal peak, and this shift has a direct impact on the match within the analysis window. However, the signal amplitude is significantly closer to the baseline values than in the case of the head. G_size = 0.773, which means an energy reduction of about 22.7%. Tolerance corridor coverage is also slightly higher than in the head, C1_corridor = 0.237, but still remains below the acceptable level. The signal temporal structure is consistent with the baseline: V_progression = 0.995, Kmax = 0.991, suggesting correct mapping of the overall waveform. The overall score C_total = 0.413 is slightly better than in the head.

The LS3 variant in the 15 g crash pulse (Figure 8c—Head, Figure 8d—Chest) presents a match profile with very high signal shape match relative to the reference signal (baseline), but with a significant temporal shift problem and partial amplitude mismatch. In the head, a time shift = +16 ms indicates a delay of the LS3 signal relative to the reference waveform by 16 ms. Such a shift results in a P_phase metric = 0.000, which, according to the CORA assessment model, signifies a lack of phase match in the defined analysis window [57]. Simultaneously, tolerance corridor coverage C1_corridor = 0.096 is highly restricted. Less than 10% of the signal falls within the expected range around the baseline waveform. This means that the signal temporal profile, although structurally similar, is shifted enough that it ceases to be acceptable within the adopted evaluation limits. The waveform amplitude remains moderately lower than the reference (G_size = 0.848), corresponding to a reduction in dynamic energy of approximately 15.2%. Despite these mismatches, the curve shape analysis indicates very strong correlation (Kmax = 0.9948) and waveform consistency (V_progression = 0.9974). The cumulative score C_total is 0.356.

For the chest, the metric values present a similar pattern. Time shift = +29 ms signifies a significant delay, which also results in P_phase = 0.000. The value C1_corridor = 0.213 suggests that only about 21% of the LS3 signal is within the permissible corridor. Despite this, the amplitude is very close to the reference (G_size = 0.899), and the structural match remains at a high level (V_progression = 0.994, Kmax = 0.9888). The overall score C_total = 0.422 remains low, but better than in the head, mainly due to better amplitude match and slightly greater corridor coverage.

The LS3 variant in the 15 g crash pulse is characterized by very good signal shape mapping quality (Kmax > 0.98 for both areas), but only partial amplitude match (G_size < 0.9) leads to a low final score (C_total < 0.45).

The LS4 variant in the 15 g crash pulse is characterized by the best mapping quality of all three configurations relative to the reference signal, both in the head and the chest. Figure 8e and f show that LS4 maintains very good convergence with the reference waveform across the entire evaluation window. In the case of the head, a time shift = +6 ms indicates a slight delay relative to the reference signal peak. The value P_phase = 0.000 means that despite this delay, the temporal match is formally low in the CORA metric (because the delay exceeded the penalization threshold) [57]. Despite this limitation, the signal shape match remains almost ideal. V_progression = 0.9986, Kmax = 0.9972, which proves strong correlation of the LS4 waveform with the reference. The amplitude metric G_size = 0.934 means that the total dynamic energy was mapped with very little loss (~6.6%). Tolerance corridor coverage was also noticeably better than in LS1 and LS3 (C1_corridor = 0.242), although still below half. As a result, the overall score C_total = 0.443 is the highest among the variants analyzed so far for the head in this scenario.

In the chest, the LS4 configuration also presented a better match. Time shift = +11 ms is moderate, but it again results in P_phase = 0.000 due to exceeding the acceptable delay. Nevertheless, both the amplitude match (G_size = 0.938) and shape correlation (V_progression = 0.989, Kmax = 0.978) are high. Tolerance corridor match C1_corridor = 0.370 is the best of all cases analyzed so far, indicating a significant improvement in signal coverage relative to the reference waveform. The final value C_total = 0.506 is the only instance in this scenario where the overall match exceeded the 0.5 threshold.

**Figure 9:**
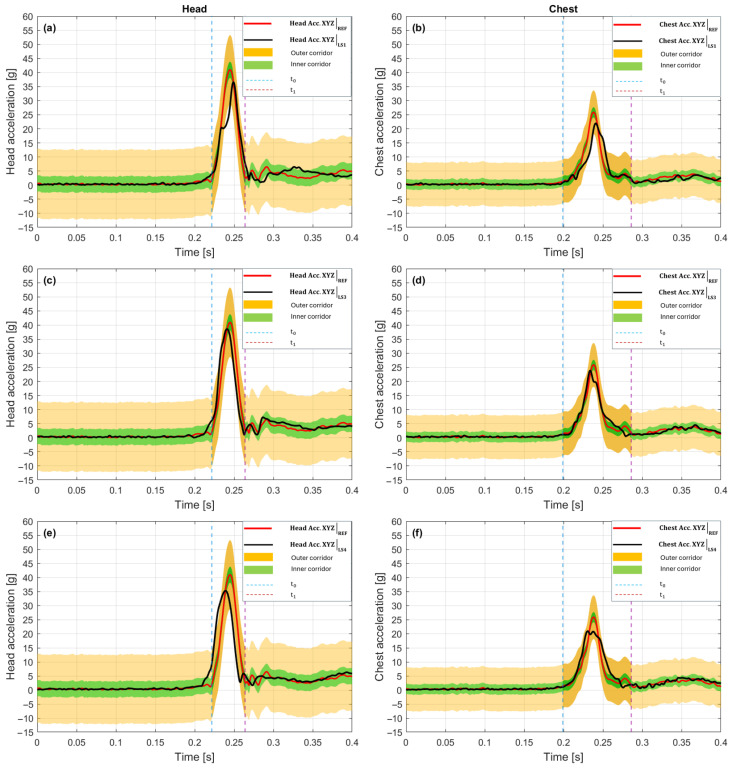
Correlation analysis of head and chest resultant accelerations for a crash pulse of 20 g: (**a**) lumbar supports variant 1 (LS1)—head; (**b**) lumbar supports variant 1 (LS1)—chest; (**c**) lumbar supports variant 3 (LS3)—head; (**d**) lumbar supports variant 3 (LS3)—chest; (**e**) lumbar supports variant 4 (LS4)—head; (**f**) lumbar supports variant 4 (LS4)—chest. The red line represents the reference signal (REF), while the black line represents the LS variant. The inner (green) and outer (yellow) corridors indicate tolerance limits. Vertical dashed lines mark the evaluation time interval [t_0_, t_1_].

The LS1 variant in the 20 g crash pulse scenario showed a clear improvement in match quality relative to previous scenarios, reflected in both the partial and final scores. In the head (Figure 9a), the time shift = +3 ms value indicates a very slight delay of the signal relative to the reference waveform. The P_phase value = 0.286 suggests that the shift is within the range where the phase score does not drop to zero, but is partially penalized. A significant improvement was achieved in tolerance corridor match. C1_corridor = 0.636, which means over 63% of the LS1 signal was within the defined evaluation range around the reference. This is a significantly higher level of match than in previous scenarios. The resultant amplitude was mapped moderately well (G_size = 0.719), corresponding to a reduction in dynamic energy of approximately 28%. Shape correlation remained very high (Kmax = 0.983, V_progression = 0.992), confirming that LS1 maps the dynamic profile with high precision. The cumulative score C_total = 0.651 qualifies as a moderately high result. In the chest (Figure 9b), this trend continued. The delay was time shift = +5 ms, which also fell within the range of partial temporal penalization (P_phase = 0.425). The C1_corridor score = 0.698 indicates that almost 70% of the signal was inside the permissible corridor, representing the best result of all previous LS1 cases. Amplitude match G_size = 0.770 suggests a slight underestimation of overloads, but without serious impact on signal quality. The waveform shape was preserved with very high accuracy (V_progression = 0.996, Kmax = 0.993), ensuring a final score C_total = 0.714.

The LS3 variant in the 20 g crash pulse achieves the best results of all previous configurations, both in terms of temporal and structural match, and also regarding amplitude mapping. For the head Figure 9c, the signal delay relative to the REF signal was practically negligible. Time shift = −3 ms, resulting in a relatively high temporal score, P_phase = 0.286.

The LS3 waveform coverage relative to the tolerance corridor was C1_corridor = 0.532, meaning that more than half of the signal fell within the tolerance range around the baseline waveform. Structural parameters achieved values close to the maximum: Kmax = 0.9987 and V_progression = 0.9994, confirming an almost ideal signal shape match. Amplitude match was also very high (G_size = 0.913), giving only an approximately 9% difference in dynamic energy between LS3 and the reference. The final value C_total = 0.632 classifies LS3 as a highly convergent configuration, particularly against previous results.

For the chest (Figure 9d), LS3 achieved an even higher score. Time shift = −1 ms means that the LS3 waveform was nearly synchronous with the reference. This is confirmed by the phase score P_phase = 0.885, which is the highest among the cases analyzed so far. The value C1_corridor = 0.861 means that 86% of the signal fell within the tolerance corridor. This is also the highest recorded result for this metric. Structurally, the waveform was still very well matched (Kmax = 0.984, V_progression = 0.992), and the amplitude match G_size = 0.927 suggests minimal differences in overload levels. The final value C_total = 0.898 not only exceeded the 0.80 threshold but also indicates that LS3 can be treated as a reference variant for this scenario.

In the LS4 configuration for the 20 g crash pulse, a clear discrepancy in match quality was noticeable between the head and the chest. Although a very good structural signal match was maintained in both cases, the results of the C_total metric differ depending on the sensor location. For the head (Figure 9e), time shift = −6 ms indicates a slight lead of the LS4 waveform relative to the REF signal. However, P_phase = 0.0 suggests that this shift exceeded the temporal metric penalization threshold, which lowered the final score. Simultaneously, V_progression = 0.998 and Kmax = 0.996 prove almost ideal signal shape match, confirming that temporal misalignment is the only serious factor limiting the final score. Tolerance corridor coverage C1_corridor = 0.278 was relatively low, which was partially due to the earlier occurrence time of the peak, causing the LS4 signal to fall outside the main evaluation window.

Amplitude was mapped well, with G_size = 0.859, meaning a difference of around 14% relative to the reference energy. The overall value C_total = 0.448 is classified as an intermediate result, dominated by high waveform correlation and acceptable amplitude mapping. For the chest (Figure 9f), the situation was significantly more favorable. Temporal shift was eliminated (time shift = 0 ms), resulting in a P_phase score = 1.0 (full temporal match). Tolerance corridor coverage was high. C1_corridor = 0.710, meaning that over 70% of the LS4 signal fell within the defined tolerance range around the reference waveform. Amplitude match G_size = 0.929 and correlation Kmax = 0.962 (with V_progression = 0.981) prove high-quality mapping in terms of both energy and dynamic structure. The final value C_total = 0.840 exceeds the 0.80 threshold, classifying this case as technically very good and close to the reference configuration.

Comparing the results of variants LS1, LS3, and LS4 across different crash pulses, clear differences in the quality of mapping the model’s dynamic response relative to the reference waveforms were observed, both in the head and the chest (Table 7). The LS1 variant was characterized by significant variability between crash pulses. In the 10 g crash pulse, it exhibited low C_total values (0.287 for Head, 0.413 for Chest), mainly due to significant temporal shifts (time shift = +12 ms and +23 ms), which resulted in total lack of phase match (P_phase = 0.000) and low tolerance corridor coverage (C1_corridor < 0.24). Despite a high shape match (Kmax > 0.98), a low level of dynamic energy (G_size close to 0.2) further lowered the final score. In subsequent crash pulses, this quality gradually improved: in the 20 g crash pulse, time shift decreased to +3/+5 ms, P_phase increased (to 0.286 and 0.425), and C1_corridor reached values above 0.63. Ultimately, LS1 in the 20 g crash pulse achieved C_total = 0.651 for Head and 0.714 for Chest, indicating a clear improvement in mapping, particularly in terms of synchronization and corridor coverage.

## 4. Discussion

The results of the conducted research clearly indicate that the use of lumbar overlays significantly affects the dummy’s biomechanical response, where this effect is closely linked to the characteristics of the crash pulse. The analyzed overlays, which differed in both size and mounting position on the seatback, substantially modified the dummy–seat interaction. As part of this work, the most detailed possible qualitative and quantitative comparison of head and chest acceleration traces was performed, comparing the LS configurations with the reference system (REF). The quantitative analysis included, among others, the determination of peak values and their times of occurrence, percentage changes compared to the reference configuration, and the calculation of the Root Mean Square Error (RMSE) in several variants (RMSE plain, aligned, and scaled, including local analysis), which allowed for the separation of the influence of amplitude differences, phase shifts, and the shape of the traces themselves on the observed deviations from the baseline configuration. Additionally, the CORA methodology was applied to determine the metrics, which enabled a quantitative assessment of signal correlation in terms of remaining within the tolerance corridor, temporal synchronization, and acceleration level. The combination of RMSE and CORA analysis results with the qualitative assessment of the trace shapes allowed for a detailed evaluation of the biomechanical response depending on the specific overlay type and for a precise determination of the changes in the dummy’s movement kinematics resulting from the use of these accessories.

In response to rear impact, the mechanical properties of the seat system (compliance, damping, hysteresis) and the timing of achieving stable support are of key importance. Stable support is defined as the moment from which loads are transferred along a continuous path, without free play or slippage, and the kinematics of the segments become synchronized. The backrest operation occurs within a “working window”: first, rapid, elastic deflection closes tolerances and initiates support, then controlled deformation dissipates the crash pulse without significant phase disturbance. Differences are observed depending on the crash pulse level: for low crash pulse levels, amplitude scaling dominates while the waveform shape is preserved; for medium crash pulse levels, timing has a greater impact (temporal shifts, segment synchronization); and for high crash pulse levels, controlled deformation prevails, enabling the reduction in peak values without significant phase shifts. In the analysis, the influence of scale and phasing is distinguished by adjusting time (time shift) and amplitude and by evaluating the match metrics (P_phase, V_progression, Kmax, RMSE) determined for the Head/Chest signals.

It is important to note that the observed delays in the dummy’s response may result not only from material compliance but also from unquantified geometric changes in the initial posture (such as increased backset) induced by the additional support volume. However, it should be emphasized that a standardized dummy positioning procedure was strictly applied in every test to minimize the risk of radical postural variability. Nevertheless, inherent geometric shifts caused by the physical presence of the overlay could not be entirely excluded.

At a low crash pulse (10 g), the overlays primarily affect the scale of the response, while maintaining the general shape of the waveforms. For LS1 and LS4, the temporal shifts in the first maximum are small (towards later and earlier occurrence, respectively), whereas LS3 is characterized by a distinct peak delay in both the head and the chest. This is consistent with the observation that with undeteriorated geometry and backset, compliance itself rarely changes the temporal shift, apart from situations related to the delay of stable support [8,29,47].

In LS1 (for the head and the chest), there was an amplitude reduction compared to the reference configuration (REF). In the head, the maximum appears later, and in the chest, it appears earlier. This pattern indicates a local “softening” of contact without geometry change. The differences between LS1 and REF result mainly from a change in signal scale, not its shape or sequence of events [29]. In LS3 (Head and Chest), the amplitude was lower than in REF, and the first maximum occurred distinctly later in both segments. The onset profile was stretched in time, indicating a slower achievement of stable support while the headrest geometry was preserved. Such delayed phasing, associated with a change in the compliance of intermediate layers, is described in the literature [8]. In LS4 (Head and Chest), the head response remains similar in scale to the reference and appears slightly earlier, whereas in the chest, the amplitude was lower, and the maximum occurred later. This supports the hypothesis that “medium” seatback stiffness can limit peak values (especially in the chest) while maintaining temporal order in the head response [29,47].

Comparing the chest dynamics with the head response: in LS1, an earlier maximum in the chest was noted with a simultaneous reduction in scale, while in LS3 and LS4, the chest maxima appeared later than in REF. Differences between LS and REF are primarily scaling in nature; the shape and sequence of events remained similar, and phase deviations were most visible in LS3 [29,43,58]. After temporal shift adjustment (time shift) and amplitude rescaling, the discrepancies further decreased; high CORA matches (V_progression and Kmax) with occasional phase drops (mainly LS3) are typical for minor time corrections [9,45,46].

The peak moment is determined primarily by headrest and backset geometry and the timing of stable support. The “softness” of the contact itself has a limited impact on temporal shift, which explains the small shifts in LS1 and LS4 and the clear delay in LS3 (interpreted as later stabilization of support) [8,47]. Excessive compliance can delay contact and increase relative neck motion, which has been associated with less favorable kinematics [2,30]. Solutions like WHIPS/SAHR (Saab Active Head Restraint) show that controlled deformation and “medium” seatback stiffness reduce peaks without phase disturbance, which corresponds well with LS1 Head and Chest and LS4 (Head), and the deviation in LS3 is linked to the support delay [7,50,51,59,60].

The response waveform is determined primarily by the timing of contact and the effective backset. Delayed achievement of stable torso support by the backrest shifts the response in time and favors an increase in the head maximum (consistent with reports regarding excessive compliance and enlarged backset) [8,32,34]. The opposite arrangement (smaller effective backset and faster, stable support) limits phase shifts and reduces neck injury criteria (e.g., NIC and Nkm), which translates into a more favorable head response amplitude and preservation of the event sequence, given improved headrest geometry and earlier support [8,9].

A variant providing earlier and more stable support (interpretively analogous to LS1) reduces the peak magnitude even when the culmination moment itself occurs later relative to the reference, because the system absorbs the crash pulse in a coordinated manner (head and torso supported parallelly) before adverse relative motion develops [50,51]. This mechanism is fundamental to controlled deformation solutions (WHIPS/SAHR): they shorten the time to support and synchronize motion, reducing peak values without redefining the waveform shape [7]. A similar philosophy is realized by “timing” approaches (sliding/energy-absorbing systems), which record drops in neck injury criteria when the phase and stability of support improve [39].

In the alignment metrics (CORA aligned), the pattern manifests itself by a decrease in P_phase with positive time corrections (time shift), while simultaneously maintaining a high shape and progression correlation (V_progression, Kmax close to 1), the signature of ‘the same curve shifted in time,’ typical for excessive compliance and degraded geometry [2,8,30]. Overall, the differences between variants in the average crash pulse confirm the hypothesis ‘geometry and timing over compliance alone’: systems with earlier and stable support perform favorably in terms of amplitude and phase, whereas overly compliant solutions without geometry control amplify peaks and worsen the phase [8,9,32,34,39,51].

The overall picture indicates that all three inserts reduce peak values in the head and chest without significant phase shifts—a typical signature of controlled deformation with preserved temporal alignment [7,51,60]. The mechanism can be described as the seatback’s ‘operating window’: the crash pulse is absorbed and dissipated in two phases, and the head restraint geometry remains synchronized, thereby preventing delayed contact; such a regime has been reported for medium stiffness solutions (WHIPS/SAHR) [7,50,51,60]. In the alignment metrics, this manifests as high temporal alignment (P_phase) and small time corrections (time shift), which confirms that the differences between the traces result primarily from a change in magnitude rather than a change in shape of the acceleration signal—the same curve with a lower amplitude [4]. This contrasts with the excessive compliance regime, where delayed contact favors an increase in peaks; here, medium stiffness reduces the amplitudes without violating the signal morphology [47].

Alternative solutions focused on timing—energy-absorbing systems/sliding seat base—also lead to a decrease in neck injury criteria, provided they achieve an earlier, gentle load transfer and maintain event synchronization [39]. The expected temporal effect of controlled solutions (described for WHIPS/SAHR) is a shorter time to stable support compared to conventional structures, without phase degradation; the observed lack of significant time shift in S3 is consistent with this and indicates stabilized geometry and rapid support [39,50,51]. From a material perspective, the moderate stiffness range of foams and hysteresis control favor amplitude damping without prolonging the reaction time [2,30]. Coupling in the pelvic area also plays a significant role: lumbar inserts modulate the pelvis–seatback connection; overly compliant hip support delays the coupling and worsens head kinematics, whereas optimal flexibility absorbs energy without sacrificing early, stable support [32,58]. The consistency of this picture is confirmed by metrics after time and scale alignment: low RMSE and high V_progression and Kmax indicate preservation of the curve shape with differences resulting mainly from amplitude, expected in the controlled deformation regime [39,50,51,61].

With the 10 g crash pulse, the overlays mainly scale the amplitudes without significant changes in the sequence of events: the configuration similar to LS1 lowers peaks with minimal phase changes, LS4 dampens peaks (especially in the chest) while maintaining chronology, and LS3 indicates a later establishment of support. In Crash pulse 2 (15 g crash pulse), the contact timing and effective backset determine the trace: earlier, stable support synchronizes the movement of the head and torso and limits peaks; greater compliance or an increased backset shifts the curves in time and amplifies the amplitudes. In Crash pulse 3 (20 g crash pulse), all overlays reduce peak values without significant phase shifts, which corresponds to predictable, controlled deformation in the defined ‘operating window’.

Design decisions should balance comfort and safety. If comfort increases due to the addition of a soft layer, this should be compensated for by a stiffer core or a reduced thickness of the overlay. If the backset increases as a result of using an overlay, it is advisable to move the entire head restraint closer or reduce the thickness of the overlay in the occipital zone. If the rebound phenomenon is detected to intensify, the material damping should be increased, or a retention element limiting the bounce should be added.

Considering the experimental results and global literature data, continuation of research towards determining injury criteria is justified. The current analysis primarily focuses on comparing head and chest acceleration waveforms and assessing signal shape differences between the reference configuration and the seat equipped with various lumbar supports. While this allows for describing the influence of the supports on the biomechanical response of the Hybrid III dummy, it does not directly translate into an injury risk assessment. From a safety engineering perspective, it is crucial not only to determine that a given configuration alters the amplitude and timing of acceleration peaks, but also to establish whether the observed modifications lead to a decrease or increase in the probability of head, neck, or chest injuries, expressed via injury criteria defined for the Hybrid III dummy. Consequently, the next stage of research should involve linking the recorded waveforms with appropriate injury indices, analyzing their sensitivity to changes in the crash pulse, as well as the type and stiffness of the lumbar supports. Furthermore, where possible, reference should be made to available data from crash tests and real-world accident databases. This approach will enable the transition from a “pure” analysis of dummy biomechanics to a quantitative assessment of injury risk and the formulation of practical recommendations regarding the safe use of lumbar supports and potential changes in safety assessment procedures.

## 5. Conclusions

This study analyzed the influence of accessory lumbar supports on the biomechanical response of the Hybrid III (50th percentile male) dummy during rear-end collisions. A series of sled tests was conducted under laboratory conditions using crash pulses of 10 g, 15 g, and 20 g, comparing a reference seat with configurations equipped with three types of supports (LS1, LS3, LS4). To assess discrepancies in head and chest acceleration waveforms, objective curve correlation methods (CORA) and Root Mean Square Error (RMSE) analysis were applied. Based on the obtained results, the following conclusions were formulated:

- Measurable biomechanical deviations. It was demonstrated that ergonomic lumbar supports introduce measurable deviations in the dummy’s response regarding passive safety. They modify the dummy-seat interaction, altering acceleration characteristics to a degree dependent on the collision energy.
- Damping and delay mechanism. The introduction of supports leads to a reduction in peak acceleration values (damping effect), which theoretically may be beneficial. However, this is accompanied by an unfavorable phase shift; maximum loads occur with a delay relative to the reference configuration, extending the duration of lack of contact between the head and the head restraint.
- Differentiation of support characteristics. Individual types of supports exhibited distinct behavior. The LS1 model maintained the highest phase consistency with the reference seat, differing mainly in signal amplitude. Conversely, the LS3 model introduced significant temporal distortions and delays (exceeding 40 ms), resulting in low correlation indices. The LS4 model exhibited intermediate properties.
- Nature of signal discrepancies. Detailed analysis using the RMSE method showed that for stable supports (such as LS1), differences result mainly from the scaling of physical quantities (amplitude), whereas for more compliant supports (such as LS3), the dominant error is the time shift and change in signal shape. This suggests the necessity of including comfort-enhancing accessories in comprehensive vehicle safety analyses.

The presented study constitutes a foundational analysis of the kinematic interaction between accessory lumbar supports and the occupant, yet the topic remains open for further investigation. Future research should extend the scope to include other types of comfort accessories, specifically seat pan cushion overlays and headrest supports. A critical area for further investigation is the analysis of the interaction and synergistic effects of multiple overlays used simultaneously. Furthermore, future work should aim to develop mathematical models that quantitatively describe how the specific changes in the occupant’s initial position, induced by these add-ons, correlate with the resulting deviations in the biomechanical response.

## Figures and Tables

**Table 1:** Summary of peak acceleration values and temporal shifts for the head and chest under a 10 g crash pulse; REF—reference configuration, LS—lumbar support.

Configuration	Peak Head [g]	Δ% Head vs. REF	t_Peak Head [s]	Δt Head [ms]	Peak Chest [g]	Δ% Chest vs. REF	t_Peak Chest [s]	Δt Chest [ms]
REF	24.88	0	0.249	0	12.71	0	0.254	0
LS1	9.34	−62.5	0.269	20	7.55	−40.6	0.234	−20
LS3	19.37	−22.2	0.297	48	11.47	−9.8	0.297	43
LS4	25.34	1.8	0.245	−4	11.32	−10.9	0.270	16

**Table 2:** Summary of peak acceleration values and temporal shifts for the head and chest under a 15 g crash pulse; REF—reference configuration, LS—lumbar support.

Configuration	Peak Head [g]	Δ% Head vs. REF	t_Peak Head [s]	Δt Head [ms]	Peak Chest [g]	Δ% Chest vs. REF	t_Peak Chest [s]	Δt Chest [ms]
REF	30.41	0	0.238	0	17.75	0	0.231	0
LS1	20.27	−33.34	0.263	0.025	14.37	−19.06	0.246	0.015
LS3	34.57	13.69	0.254	0.016	19.65	10.68	0.254	0.023
LS4	32.39	6.51	0.244	0.006	15.93	−10.28	0.237	0.006

**Table 3:** Summary of peak acceleration values and temporal shifts for the head and chest under a 10 g crash pulse; REF—reference configuration, LS—lumbar support.

Configuration	Peak Head [g]	Δ% Head vs. REF	t_Peak Head [s]	Δt Head [ms]	Peak Chest [g]	Δ% Chest vs. REF	t_Peak Chest [s]	Δt Chest [ms]
REF	40.98	0	0.245	0	25.88	0	0.238	0
LS1	36.67	−10.53	0.249	0.004	21.99	−15	0.241	0.003
LS3	38.66	−5.67	0.241	−0.004	23.95	−7.46	0.234	−0.004
LS4	35.26	−13.97	0.239	−0.006	20.89	−19.27	0.231	−0.007

**Table 4:** Comparison of Root Mean Square Error (RMSE) metrics before and after amplitude matching for the 10 g crash pulse scenario; LS—lumbar support.

Segment	LS	RMSE Aligned [g]	RMSE Scaled [g]	Change After Matching [%]	Qualitative Interpretation
Head	LS1	0.79	0.54	−31.6	very good match after amplitude correction
	LS3	0.91	0.63	−30.8	correct shape, amplitude lower
	LS4	0.85	0.59	−30.6	moderate improvement, lower intensity
Chest	LS1	1.02	0.74	−27.5	best fit in the analyzed area
	LS3	1.10	0.81	−26.4	larger error, required stronger amplitude correction
	LS4	1.05	0.78	−25.7	good match, waveform slightly weaker than the baseline

**Table 5:** Comparison of Root Mean Square Error (RMSE) metrics before and after amplitude matching for the 15 g crash pulse scenario; LS—lumbar support.

Segment	LS	RMSE Aligned [g]	RMSE Scaled [g]	Change After Matching [%]	Qualitative Interpretation
Head	LS1	0.83	0.58	−30.1	very good match after amplitude correction
	LS3	1.04	0.72	−30.8	largest error, differences in amplitude and time
	LS4	0.91	0.63	−30.8	good match, amplitude slightly lower
Chest	LS1	1.15	0.83	−27.8	best fit in the analyzed area
	LS3	1.26	0.95	−24.6	larger error, signal less faithfully mapped
	LS4	1.19	0.88	−26.1	good match, moderate amplitude differences

**Table 6:** Comparison of Root Mean Square Error (RMSE) metrics before and after amplitude matching for the 20 g crash pulse scenario; LS—lumbar support.

Segment	LS	RMSE Aligned [g]	RMSE Scaled [g]	Change After Matching [%]	Qualitative Interpretation
Head	LS1	0.92	0.68	−26.1	best fit, very good match with the baseline
	LS3	1.18	0.84	−28.8	largest error, differences in amplitude and time
	LS4	1.01	0.73	−27.7	good match, amplitude slightly reduced
Chest	LS1	1.22	0.90	−26.2	lowest error, correct shape mapping
	LS3	1.34	1.03	−23.1	largest discrepancies, required stronger amplitude correction
	LS4	1.28	0.95	−25.8	good match, moderate difference in signal strength

**Table 7:** Quantitative comparison of model dynamic response quality across different crash pulse intensities (10–20 g) based on correlation analysis; LS—lumbar support.

LS	Crash Pulse	Part	C_Total	C1_Corridor	V_Progression	P_Phase	G_Size	Kmax	T_Shift
LS1	10	Chest	0.592	0.679	0.996	0.172	0.347	0.991	−12
		Head	0.406	0.422	0.993	0	0.176	0.987	18
	15	Chest	0.413	0.237	0.995	0	0.773	0.991	23
		Head	0.287	0.168	0.991	0	0.228	0.982	12
	20	Chest	0.714	0.698	0.996	0.425	0.77	0.993	5
		Head	0.651	0.636	0.992	0.286	0.719	0.983	3
LS3	10	Chest	0.574	0.508	0.992	0	0.927	0.983	53
		Head	0.463	0.467	0.994	0	0.381	0.989	42
	15	Chest	0.422	0.213	0.994	0	0.899	0.989	29
		Head	0.356	0.096	0.997	0	0.848	0.995	16
	20	Chest	0.898	0.861	0.992	0.885	0.927	0.984	−1
		Head	0.632	0.532	0.999	0.286	0.913	0.999	−3
LS4	10	Chest	0.595	0.567	0.994	0	0.876	0.989	15
		Head	0.622	0.505	0.997	0.306	0.913	0.994	−5
	15	Chest	0.506	0.37	0.989	0	0.938	0.978	11
		Head	0.443	0.242	0.999	0	0.934	0.997	6
	20	Chest	0.84	0.71	0.981	1	0.929	0.962	0
		Head	0.448	0.278	0.998	0	0.859	0.996	−6

## Data Availability

The raw data supporting the conclusions of this article will be made available by the author on request.

## Abbreviations

The following abbreviations are used in this manuscript:

ATD	Anthropomorphic Test Device
BioRID	Biofidelic Rear Impact Dummy
CORA	CORrelation and Analysis
LS	Lumbar Support
NIC	Neck Injury Criterion
Nkm	Neck protection criterion
REF	Reference
RMSE	Root Mean Square Error
SAHR	Saab Active Head Restraint
WAD	Whiplash Associated Disorders
WHIPS	Whiplash Protection System
